# Factors Influencing Learner Permit Duration

**DOI:** 10.3390/safety3010002

**Published:** 2016-12-22

**Authors:** Johnathon P. Ehsani, Kaigang Li, Brydon J. B. Grant, Pnina Gershon, Shelia G. Klauer, Thomas A. Dingus, Bruce Simons-Morton

**Affiliations:** 1 Center for Injury Research and Policy, Department of Health Policy and Management, Johns Hopkins Bloomberg School of Public Health, Baltimore, MD 21205, USA; 2 Department of Health and Exercise Science, Colorado State University, Fort Collins, CO 80523, USA; 3 Department of Epidemiology and Environmental Health, State University of New York at Buffalo, Buffalo, NY 14214, USA; 4 Health Behavior Branch, Eunice Kennedy Shriver National Institute of Child Health and Human Development, Bethesda, MD 20817, USA; 5 Virginia Tech Transportation Institute, Blacksburg, VA 24061, USA

**Keywords:** novice teenage drivers, parents, learner permit, licensure

## Abstract

An increasing number of countries are requiring an extended learner permit prior to independent driving. The question of when drivers begin the learner permit period, and how long they hold the permit before advancing to independent licensure has received little research attention. Licensure timing is likely to be related to “push” and “pull” factors which may encourage or inhibit the process. To examine this question, we recruited a sample of 90 novice drivers (49 females and 41 males, average age of 15.6 years) soon after they obtained a learner permit and instrumented their vehicles to collect a range of driving data. Participants completed a series of surveys at recruitment related to factors that may influence licensure timing. Two distinct findings emerged from the time-to-event analysis that tested these push and pull factors in relation to licensure timing. The first can be conceptualized as teens’ motivation to drive (push), reflected in a younger age when obtaining a learner permit and extensive pre-permit driving experience. The second finding was teens’ perceptions of their parents’ knowledge of their activities (pull); a proxy for a parents’ attentiveness to their teens’ lives. Teens who reported higher levels of their parents’ knowledge of their activities took longer to advance to independent driving. These findings suggest time-to-licensure may be related to teens’ internal motivation to drive, and the ability of parents to facilitate or impede early licensure.

## 1. Introduction

Informal practice constitutes an important element of learning to drive [1]. Some countries, such as New Zealand, have required an extended learner period for almost 20 years [2]. However, a large number of countries still do not have, or have only recently required a learner permit period, as part of a staged licensing system [3,4]. There is an increasing recognition that the learner stage represents a valuable opportunity for novice drivers to develop the skills and safety judgments necessary [5–7] to reduce the very high crash risk corresponding to the first few months of independent driving [8].

Older teenagers are somewhat safer when they begin to drive independently than those who are younger [3] (due presumably to greater maturity or self-selection). An extended period of practice during the learner stage has been shown to confer a safety benefit during independent driving [9]. Therefore, the question of when drivers begin the learner permit period, and how long they hold the permit before advancing to independent licensure, takes on a special significance. The limited research on this subject suggests the path to licensure is not determined exclusively by the safety considerations of the learners or the supervisors.

There may be several “push” and “pull” factors influencing licensure timing and the duration of the learner license period. In the United States and elsewhere, teens cite factors that pull them back from getting licensed such as the cost associated with driving, the ability to get around without a car, and being busy with out-of-school activities as reasons for licensure delay [10–12]. In contrast, some teens are highly motivated (pushed) to begin driving and obtain their learners’ permit within days of reaching eligibility [13]. These teens will typically have access to their own vehicle when they begin driving, or have part-time employment which requires them to drive [10,14]. It is likely that teens are not only pushed or pulled, but these factors vary within teens, they may interact, and may change overtime.

Among those who begin the formal licensing process, relatively little is known about the variability in the duration the learner permit is held (beyond the minimum number of months required by Graduated Driver Licensing) and the amount of practice that is accumulated during this period. An improved understanding about why some teens advance quickly through the learner permit period and others take considerably more time, may inform our understanding of how to reduce the high crash risk during the independent driving stage. Previous studies on licensure timing have been cross-sectional, using self-reported surveys and comparing the characteristics of those who are licensed earlier to those who are licensed later [10,13] but have not examined the factors influencing progression through the licensing process, among those who have obtained their learner permit. Similarly, the literature on parent and teen engagement during the accompanied driving period has described the factors related to the experience of practice driving, such as social support [15], parental modeling of driving behavior [16] and the pre-existing parent-teen dynamic [17], but do not examine how these factors influence the duration of the practice driving period.

**Figure 1:**
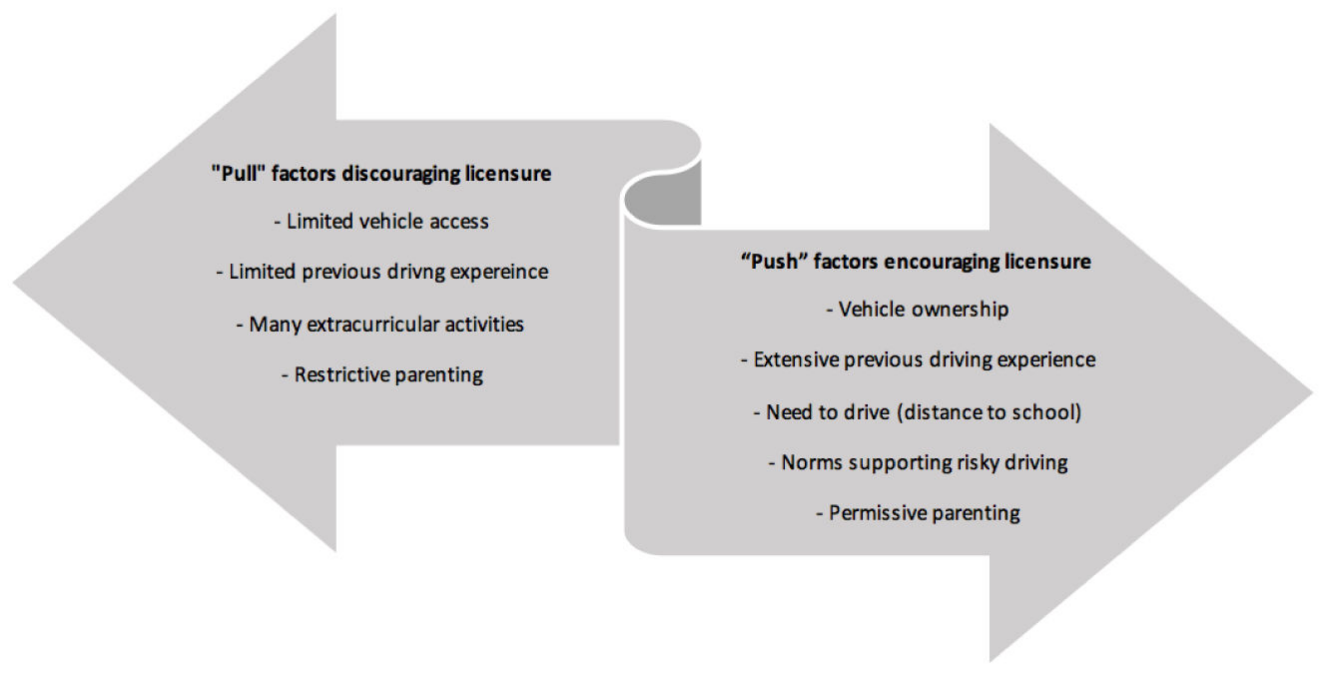
Factors encouraging or discouraging independent licensure.

We recruited a sample of novice drivers soon after they obtained a learner permit and instrumented their vehicles to collect a range of driving data. In this paper, we report the variability in novice teenage drivers’ learner permit duration according to various “push and pull” factors (Figure 1). “Push” factors correspond to individual, psycho-social and environmental factors encouraging novice teenage drivers to advance through the learner permit period. It is hypothesized push factors would encourage teens to obtain their learner permit as close to their date of eligibility as possible and advance to independent licensure shortly after fulfilling the requirements of the minimum holding period. These factors could include needing to drive in order to get to school, or having friends who are pursuing licensure or are already driving [10]. “Pull” factors which discourage teens from independent licensure could include limited access to a vehicle, a lack of time to practice [11,12], or low levels of parental trust towards their teen [18] which might constrain teens’ ability to practice.

Theoretically, delayed licensure would confer possible safety benefits owing to older age at licensure and potentially greater driving experience. However, perhaps not all learners need the same amount of practice driving and there may be practical reasons for relatively early licensure [19]. The purpose of this study was to examine the contribution of factors that may encourage teens to advance quickly to independent licensure, or impede them and result in a longer learner permit period, using a combination of surveys and naturalistic driving methods.

## 2. Materials and Methods

The primary vehicles of learner teenage drivers in southwestern Virginia, USA were instrumented with data acquisition capabilities within three weeks of obtaining a learner’s permit. These data acquisition systems were used for the purpose of measuring amount of practice completed before progressing to independent licensure. Participants were instructed to drive as they would normally.

### Participants and selection criteria

The study required the participation of teenage drivers and at least one of their parents. Recruitment was conducted in local newspapers and high schools in southwestern Virginia, USA. Teen participants were initially screened in a telephone interview for eligibility, using the following inclusion criteria: (a) being between 15.5 and 16.1 years old; (b) holding for no more than three weeks a learner driver’s license allowing supervised driving; (c) having at least 20/40 corrected vision; (d) having at least one parent willing and able to participate; (e) access to a vehicle expected to survive mechanically for at least 18 months; (f) residing within a one-hour drive of the research center or satellite location; and (g) holding liability insurance on the vehicle to be used in the study (required by state law).

Participants were excluded based on the prescreen telephone interview if they: (a) had a diagnosis of attention deficit disorder (ADD) or attention deficit hyperactivity disorder (ADHD); (b) had an identical twin (which would make it difficult to distinguish participants during coding); and (c) needed to enter restricted areas (i.e., that do not allow cameras for security reasons.

Participant recruitment was stratified to have a similar number of male and female teenage drivers. A total of 298 individuals responded to recruitment efforts, of which 90 fulfilled the eligibility criteria and were enrolled in the study. The final teenage sample comprised 49 females and 41 males with an average age of 15.6 years (Standard Deviation 0.2). Participant age at obtaining their learner permit was determined by requesting an official form of identification that included birthdate.

Three consent forms were required for the study: parental consent and teenagers’ assent for their participation, and an adult consent form for parent participation. In 41 families, two adults were consented for the study. Teenager assent was obtained separately from the parent to ensure their participation was voluntary, and free of parental coercion. Teenage participants received $800 for completing the study, paid to them in installments as they completed key milestones. The protocol was approved by the Virginia Tech Institutional Review Board for the Protection of Human Subjects.

The data acquisition system installed in the vehicles included a computer that received and stored continuous data from vehicle sensors, including accelerometers and a global positioning system (GPS) that calculated vehicle position. Trip duration (in minutes) was calculated using the timestamp corresponding to ignition on and ignition off, regardless of vehicle movement. GPS recorded the movement of the vehicle and the distance traveled in each trip. Practice driving hours and miles were derived from these values.

Thirteen participants’ driving data was collected from two vehicles, and a single participant provided data from three vehicles. The majority of supervising parents enrolled in the study were females (63.3%). Approximately half the sample (46.6%) reported a household income of over $100,000. During the study period, average household income in Virginia was $61,406 [20]. Data were collected from January 2011 to August 2014. Participants were followed for the duration of their learner permit period, for a minimum of 24 months.

### Outcome Measure

Licensure timing of participants was determined by requesting a copy of the participants’ driver’s license when they notified the research team when they had progressed to their independent driving license. The duration of the learner permit period was calculated by subtracting the date of independent licensure from the date the learner permit was issued.

### Predictors of Licensure Timing

Surveys were administered at baseline and included measures of individual characteristics and behaviors, perceptions of peer norms and parental restrictions that could be considered as “push or pull” factors encouraging or discouraging the progression to independent licensure. The scales administered for this study are as described below:

- *Pre-permit driving experience:* Participants were asked eight questions related to their first time driving different vehicle types, and the number of times they had driven each type. Vehicle types included cars/trucks, all-terrain vehicles, motorcycles/scooters, boats, jet skis, golf carts, tractors and riding lawn mowers. The frequency of driving each vehicle type ranged from never to five or more times. The cumulative driving experience was averaged across all vehicle types. We hypothesized that greater driving experience prior to obtaining a learner permit may reflect a higher interest in driving and, therefore a greater motivation to advance to independent licensure.
- *Sensation seeking:* The Hoyle brief sensation seeking measure [21] asks participants to rate their attitude towards eight statements relating to thrill and adventure seeking, experience seeking, disinhibition and boredom susceptibility. Responses were made on a true and false scale. Teens who score highly in sensation seeking may be more motivated to drive, and advance to independent licensure sooner than those who are less motivated to drive.
- *Distance from school:* A single multiple-choice question measured participants’ distance from their school, as a proxy measure of their need to drive. Participants could choose a single option from the following distances: less than 1 mile, 1 to 5 miles, 6 to 10 miles, more than 10 miles. Greater distance to school may result in a greater need to drive. We hypothesized that teens who lived further from school would advance to independent licensure sooner than those who lived in closer to their school.
- *Friends’ risky driving:* This sub-scale of Aker’s measure [22] included 10 statements assessing how participants perceived their friends’ risky driving behaviors. Participants were asked to rate their friends’ behavior on a scale of 1 = *None* to 5 = *All,* how many of their friends followed road rules carefully, exceeded speed limits, drove aggressively, engaged in secondary tasks or drove after using marijuana or drinking alcohol, among other items. Having friends who are engaging in risky driving may reflect a peer context where driving is encouraged, which may result in a shorter time to advance to independent licensure.
- *Expected driving privileges:* Thirteen items measured participants’ expected driving privileges within the first 3 months of independent driving. Participants completed a 10-point scale ranging from 1 = *never* to 10 = *very frequently* did they expect to be allowed to drive under different conditions such as with peer passengers, on high speed roads, late at night, or without telling a parent where they are going.
- *Parental trust of teen:* This 6-item measure of perceived parental trust was adopted from Simons-Morton et al. [23], and assessed how much participants perceived their parents trusted them. Items ranged from general concepts: “How much do your parents trust that you will not hang out with bad people?” to specific situations: “How much do your parents trust what you say you are doing to do on a Saturday night is true?”. Response ranged from 1 = *Not at all* to 4 = *A lot*. Perceptions of parental trust may reflect a teen’s autonomy and independence. We hypothesized that teens who reported perceiving higher levels of parental trust would advance sooner to independent licensure.
- *Out-of-school activities:* The amount of time participants spent on out-of-school activities was measured using two items: (1) how many days a week do you spend on these activities (ranging from 0 to 7) and (2) on a typical day, how many minutes do you spend on these activities (which was an open ended item). The values from the two measures were multiplied to quantify the number of minutes spent on these activities each week. We hypothesized that teens involved in more out-of-school activities may have less time for practice driving and therefore take longer to advance to independent licensure.
- *Parental Knowledge of Teen Activities:* This measure included eight items relating to teens perceptions of their parental knowledge about their activities, adapted from Simons-Morton et al. [23]. For different behaviors, teens were asked to state “How often do your parents know?” with response options ranging from 1 = *Never* to 5 = *Always*. Examples of items questions include: How much do your parents know what you do during your free time? Where you go and what you do after school?; Where you go when you are out with friends at night?; What you spend money on?. Higher perceptions of parental knowledge of their activities may reflect a greater involvement in their lives and vigilance related to risky behaviors. We hypothesized that teens who perceived parental knowledge to be high would take longer to advance to independent licensure.
- *Parental Restrictions Related to Driving:* This 13-item measure was adapted from Simons-Morton et al. [24]. It was used to assess teens’ perceptions of their driving privileges, and how they might change if their parents observed irresponsible driving behaviors. Participants completed a 7-point scale ranging from 1 = *Not at all likely* to 7 = *Extremely likely*. An example item is: “How likely is it that your parents would restrict your driving privileges if you got pulled over by the police or got a ticket?”. Perceptions of parental restriction may reflect a parenting style where teens’ irresponsible behaviors are met with consequences. We hypothesized that teens who reported having higher levels of parental restrictions would take longer to advance to independent licensure.
- *Expected Vehicle Access:* A single item measured participants’ access to a vehicle when they begin driving independently. At the beginning of the study, parents were asked if the vehicle being instrumented for the study was “going to be driven by you alone, your teen alone, or shared with your teen after they receive their license?”. We hypothesized that teens who were going to have a dedicated vehicle would advance to independent licensure sooner than teens who would share a vehicle.

### Analyses

Cronbach’s alpha coefficients were calculated to assess the internal consistency of the survey measures. Spearman correlations were calculated for the categorical and continuous push-pull factors and the duration of the learner permit period. A Wilcoxon rank-sum test was used to test the association between dichotomous variables and the learner permit duration. Cox proportional hazard regression was conducted to assess independent associations and interactions with time to licensure. Statistical analyses were performed using R.

## 3. Results

### Learner Permit Duration

In Virginia, USA, where the study was conducted, novice teenage drivers are required to hold the learner’s permit for at least nine months. Of the 90 novice teen drivers recruited for the study, 83 participants completed the learner permit stage and advanced to independent licensure. The average learner permit duration for these teens was 10.35 months (Standard Deviation (SD) = 2.48).

Three teens remained on their learner permit for the duration of the study. Four teens withdrew from the study due to vehicle-related issues (teen or parent involved in a crash and did not want to re-instrument the new vehicle, sold the instrumented car, or moved out of state). Three of the four participants withdrew before they were eligible for an independent license (i.e., before 9 months of holding their learner permit), and the fourth participant withdrew after 9.5 months, after their parent was involved in a crash and the vehicle was no longer roadworthy. None of these participants obtained their independent licensure during the study. However, the results of the bivariate and multivariate models did not change when these participants were removed or retained in the analysis. Therefore, all participants were included in the analyses presented below (see participant attributes in Table 1).

### Factors Associated with Time to Independent Licensure

The psychometric properties of the survey measures and their bivariate association with the time to independent licensure are described in Table 2. Learner permit duration was significantly negatively correlated with previous driving experience (*r* = −0.30), and parental trust of the teen (*r* = −0.25); meaning teens who had considerable driving experience prior to obtaining their learner permit, or those who perceived higher levels of parental trust, took significantly less time to progress through the learner permit stage. The remainder of the correlations between the learner permit period and the survey measures listed in Table 2 were small and not statistically significant.

The correlation between participant age and learner permit duration (not shown in Table 2) was 0.21 (*p* = 0.05), indicating that teens that were older when they began the learner stage, took significantly longer to become licensed to drive independently. Learner permit duration was not significantly associated with the number of miles (*r* = −0.16) or minutes (*r* = −0.14) participants drove during this stage of licensure, and there was no significant difference between females and males in learner permit duration. Twenty-six participants (28.9%) reported owning a vehicle, and the remainder shared a vehicle. There was no significant bivariate association between vehicle ownership and learner permit period.

### Time-to-Event Analysis of Factors Associated with Time to Licensure

Cox proportional hazard regression was conducted to assess independent associations and interactions with the time to licensure. In a multivariate model of factors influencing progression to independent licensure (Table 3), the Schoenfeld residuals test was not significant, which is consistent with the proportional hazards assumption being met.

Teens’ age when they obtained a learner permit (*p* < 0.01), and parental knowledge of teens’ (*p* < 0.05) activities were associated with later licensure, as evidenced by the hazard ratios less than 1 that indicate a longer time to independent licensure (Table 3). As teens’ age at obtaining a learner permit increased, the number of months they held a learner permit also increased. The reverse is also true; where teens who were younger when obtaining a learner permit, advanced to independent licensure sooner. As parental knowledge of teens’ activities increased, the number of months on the learner permit also increased.

Pre-permit driving experience was associated with earlier licensure (*p* < 0.01). The significant hazard ratio greater than one (1.04) indicates a shorter time to independent licensure. As teens’ pre-permit driving experience increased, the number of months they held their learner permit decreased. No significant interactions were observed in models that tested the age, the parental knowledge and the pre-permit driving experience, with the time to independent licensure.

The significant bivariate association observed between parental trust and time to independent licensure was absent in the multivariate time-to-event analysis, although the direction of the association remained the same. The variance inflation factor (VIF) was used to test for collinearity between each of the predictor variables. A VIF between 5 and 10 indicates high correlation that may be problematic [25]. The highest VIF was for parental knowledge of teens’ activities at 1.45, and the correlation coefficient between parental knowledge of teens’ activities, and parental trust was *r =* 0.43. These indicate parental trust and parental knowledge are related, but unlikely to be collinear.

## 4. Discussion

The purpose of this study was to examine the contribution of factors that push teens to advance quickly to independent licensure, or pull them back and result in a longer learner permit period. Two distinct findings emerged from the time-to-event analysis that tested these factors in relation to licensure timing. The first can be considered as teens’ motivation to drive (push), reflected in a younger age when obtaining a learner permit and extensive pre-permit driving experience. These may reflect an affinity towards cars and driving, or the presence of an enabling environment that facilitated early licensure and practice, to the extent some parents (and possibly others) allow early learner permits and (illegal) pre-learner driving. The second finding was parents’ knowledge of teens’ activities (pull); which can be considered as a proxy for a parents’ attentiveness to their teens’ lives. Teens who reported higher levels of their parents’ knowledge of their activities took longer to advance to independent driving.

Not all the push factors that have been previously described in the literature were significantly associated with licensure timing. For example, we had hypothesized that teens who had exclusive access to a vehicle are more likely to be able to practice driving, or would be more motivated (pushed) to begin driving [10]. Teen drivers who own or have access to their own vehicle are more likely to engage in a range of risky driving behaviors [26]. Therefore, the absence of an association between vehicle ownership and learner permit duration in this sample is surprising. This may be a unique characteristic of this small, non-random sample or due to measurement error. This self-reported question required parents to anticipate their teen’s access to a vehicle at least 9 months into the future. During this period, household circumstances may have changed, or parents’ experience of supervising their teen may have influenced their decision to allow unrestricted access to a vehicle.

Gender was also not associated with the length of time participants held their learner permit. Young men’s risky driving behaviors have been described extensively in the literature [27,28], and a shorter learner permit period can be considered as higher risk, and something that might “push” young men to pursue earlier licensure. The absence of an association between gender and licensure has been previously described for a sample of U.S. teens [10], which may also be reflected in this study sample.

The need to drive (measured as the distance lived from school) was hypothesized to “push” teens towards earlier licensure. However, we did not observe a significant association between the distance from school and time to licensure. This may be due to alternative means of transportation to school, or factors such as employment, that were not measured. Friends’ risky driving was also not associated with a push towards independent licensure. While risky peer norms are known to influence teens’ risky driving during independent driving [29], the absence of a peer effect on licensure timing may be due to the central role that parents play during the permit period.

A lack of time to practice has been described as a reason for delayed licensure (pull) among teens from the United States, New Zealand and the United Kingdom [10,11,14]. In this study, the time spent on out-of-school activities was not associated with the learner permit duration. This may be because participants had already initiated the process of licensure when they were recruited for the study, suggesting they had prioritized driving among the many additional demands for their time. Alternatively, while busy teens might have the greatest need to drive, they may also have more resources (parents and colleagues who could facilitate transportation) and less time.

A close parentteen relationship during the practice driving period is a pre-condition for gaining extensive practice [15–17]. However, little is known about how the decision is made for a teen to advance to independent licensure, and the role of the parent and the teen in that process. Parents facilitate access to a vehicle, provide supervision, and in many cases, pay for their teens’ progression through the expensive licensure process. Therefore, it is likely they play a critical role in deciding when the teen is ready to drive independently. This adds to the existing body of literature on the role of parents in promoting teen driver safety during the learner permit period and beyond [23,24,30,31].

The duration of the learner permit period required to develop safe driving skills and judgment is not known. The limited literature on the subject suggests that the longest possible learner period would seem best [9,32], but the minimum or optimal number of months a learner license should be held, are both unknown. Other dimensions of practice driving which were not examined in this study, such as the quality of instruction, the ability of the supervisor to calibrate lessons and practice to the emerging skills of the learner driver, and formal instruction are all likely to play a role in improving safety outcomes, although the extent to which these contribute is unknown.

We did not find that longer practice period was associated with more practice, suggesting that there may be only so much time or tolerance for practice among teens and parents. An alternative explanation is that the required number of hours may act as an anchor for the amount of practice teens accumulate [33]. This notion is supported by an Australian study where changes in the required number of practice hours (but not the learner permit duration) led to a significant increase in the amount of practice that was conducted [34]. Lengthening the permit period may not necessarily lead to more hours of practice, though it would result in older, more mature drivers. Efforts to increase the amount of practice may be more effective if they combined a longer learner permit period in combination with an increase in the amount of required hours.

## 5. Limitations

The study population was a highly motivated sample of novice drivers who had committed to ongoing assessment via surveys and naturalistic measurement of their driving behavior. They were generally from higher income households relative to the state average, and are likely to have had greater parental involvement in the process of learning to drive than the average teen in Virginia. Recruitment for this study required teens to advance through Virginia’s graduated driver licensing system, which is not required for teens who initiate driving when they are 18-years or older.

During the study period, participants completed a state-approved driver education program which is delivered by a public, private or commercial driving school. The program consists of 36 fifty-minute classroom periods of instruction and 14 fifty-minute periods of in-car instruction (seven periods of driving and seven periods of observation). The final session of driver education is the administration of the behind-the-wheel road test, which is conducted by the driver instructor. If a teen is not deemed ready to take the behind-the-wheel test, the driving instructor would discourage taking the test and suggest additional driving practice (either formal or informal). Once the teen is considered ready to take the behind-the-wheel test then the instructor will administer the test. For this reason, we did not measure when teenagers took the behind-the-wheel test and the number of test attempts.

## 6. Conclusions

We observed considerable variability in the duration of the learner permit period, and identified associations with two individual push factors, greater pre-permit driving and younger age at permit, representing a higher initial motivation to drive, and one pull factor, greater parental knowledge of teens’ activities. Taken collectively, the findings of this study suggest the duration of the learner permit period is likely to be a function of the internal motivations of the teen to begin driving, combined with an enabling or inhibiting environment influenced by parents. Parents may be particularly important sources of influence for both early and late licensure, and can discourage or accelerate advancement to licensure though clear expectations for the independent driving period.

## Figures and Tables

**Table 1:** Sample Characteristics.

Measures	Number of Participants (90)	%
Gender		
Female	49	54.44
Male	41	45.55
Race/Ethnicity		
White	82	91.11
Black	3	3.33
Other	5	5.55
Age at instrumentation		
15.5	43	47.78
15.6	21	23.33
15.7	8	8.89
15.8	8	8.89
15.9	4	4.44
16.0	4	4.44
16.1	2	2.20

**Table 2:** Psychometric Properties of Survey Scales and Correlation with Time to Independent Licensure (N = 90).

Push or Pull^	Survey Scales	Number of Items	Min	Max	Median	Mean	SD	Reliability#	Correlation with Time to Licensure
Push	Pre-permit Driving Experience	8.00	1.00	35.00	12.00	12.42	7.51	0.69	−0.30 **
Push	Sensation Seeking	8.00	1.38	4.75	2.88	2.90	0.60	0.75	−0.06
Push	Distance to School	1.00	1.00	4.00	3.00	2.76	0.86	-	−0.07
Push	Friends’ Risky Driving	10.00	1.70	3.20	2.10	2.23	0.34	0.78	−0.15
Push	Expected Driving Privileges	13.00	2.00	8.23	4.58	4.64	1.34	0.86	−0.14
Push	Parental Trust	6.00	1.00	4.00	3.67	3.49	0.55	0.84	−0.25 *
Pull	Out-of-School Activities	2.00	0.00	2000.00	480.00	527.00	354.63	-	−0.04
Pull	Parental Knowledge	8.00	2.60	4.88	4.00	4.00	0.50	0.81	0.03
Pull	Parental Restrictions Related to Driving	1.00	1.00	4.00	3.00	2.76	0.86	-	−0.07

^ Hypothesized push factors encourage earlier independent licensure and pull factors impede progress towards independent licensure;

# Standardized Cronbach Coefficient Alpha;

* *p* < 0.05,

** *p* < 0.01.

**Table 3:** Factors Influencing Time to Independent Licensure (N = 90).

Push or Pull	Individual, Peer and Parental Factors	Hazard Ratio	Confidence Interval	*p*
Push	Age at Permit	0.09	0.02–0.48	<0.01
Push	Gender	1.23	0.74–2.05	0.42
Push	Pre-permit Driving Experience	1.04	1.01–1.08	<0.01
Push	Sensation Seeking	0.92	0.64–1.31	0.63
Push	Distance to School	1.02	0.76–1.37	0.89
Push	Friends’ Risky Driving	1.38	0.66–2.85	0.39
Push	Expected Driving Privileges	0.93	0.76–1.15	0.50
Push	Parental Trust	1.29	0.73–2.31	0.38
Pull	Out of School Activities	0.99	1.00–1.00	0.72
Pull	Parental Knowledge	0.59	0.34–0.99	0.05
Pull	Parental Restrictions Related to Driving	1.00	0.81–1.24	0.98
